# Lipoprotein(a) and stroke: a two-sample Mendelian randomization study

**DOI:** 10.3389/fnagi.2023.1178079

**Published:** 2023-05-12

**Authors:** Yi Huang, Ruijie Zhang, Liyuan Han, Yiwen Wu, Xinpeng Deng, Tianqi Xu, Yuefei Wu, Xiang Gao, Chenhui Zhou, Jie Sun

**Affiliations:** ^1^Department of Neurosurgery, The First Affiliated Hospital of Ningbo University, Ningbo, China; ^2^Key Laboratory of Precision Medicine for Atherosclerotic Diseases of Zhejiang Province, Ningbo, China; ^3^Key Laboratory of Diagnosis and Treatment of Digestive System Tumors of Zhejiang Province, Hwa Mei Hospital, University of Chinese Academy of Sciences, Ningbo, China; ^4^Department of Global Health, Ningbo Institute of Life and Health Industry, University of Chinese Academy of Sciences, Ningbo, China; ^5^Department of Neurology, The First Affiliated Hospital of Ningbo University, Ningbo, China

**Keywords:** stroke, lipoprotein(a), ischemic stroke, large-artery atherosclerotic stroke, Mendelian randomization

## Abstract

**Background:**

To evaluate the causal relationship between lipoprotein(a) Lp(a) and stroke risk.

**Method:**

Adopting two grand scale genome-wide association study (GWAS) databases, the instrumental variables were selected on the basis that the genetic loci met the criteria of being independent of each other and closely related to Lp(a). Summary-level data for outcomes, ischemic stroke and its subtypes were acquired from the UK Biobank and MEGASTROKE consortium databases. Two-sample MR analyses were achieved using inverse variance-weighted (IVW) meta-analysis (primary analysis), weighted median analysis, and the MR Egger regression method. Multivariable-adjusted Cox regression models were also used for observational analysis.

**Result:**

Genetically predicted Lp(a) was marginally related with higher odds of total stroke (odds ratio (OR) [95% confidence intervals (CI)]: 1.003 [1.001–1.006], *p* = 0.010), ischemic stroke (OR [95% CI]: 1.004[1.001–1.007], *p* = 0.004), and large-artery atherosclerotic stroke (OR [95% CI]: 1.012 [1.004–1.019], *p* = 0.002) when the IVW estimator was used on the MEGASTROKE data. The associations of Lp(a) with stroke and ischemic stroke were also remarkable in the primary analysis using the UK Biobank data. Higher Lp(a) levels were also related with increased total stroke and ischemic stroke risk in the observational research data in the UK Biobank database.

**Conclusion:**

Genetically predicted higher Lp(a) perhaps rise the risk of total stroke, ischemic stroke, and large-artery atherosclerotic stroke.

## 1. Introduction

Globally, stroke deserves our attention as a major cause of disability and death (Campbell et al., 2019). And in a large, nationally representative sample of adults aged 40 Years or older, the estimated prevalence,incidence,and mortality rate of stroke in China in 2020 Were 2.6%, 505.2 per 100,000 person-years, and 343.4 per 100,000 person-years, respectively (Tu et al., 2023). Well-known risk factors include age, sex, race, genetic factors, hypertension etc. (Campbell et al., 2019). However, high levels of lipoprotein(a) Lp(a) are also an standalone risk factor for stroke (Mazhar et al., 2017). As a low-density lipoprotein-like particle, Lp(a), its serum concentration is relatively stable in individuals (Erqou et al., 2009).

Evidence have shown that high levels of serum Lp(a) can rise the stroke susceptibility (Kamstrup et al., 2009; Li et al., 2014). However, the relationship between exposure and outcome in an observational study is inevitably affected by various factors, which can lead to false correlations. The correlation between exposure and outcome may also be affected by reverse causality (Smith and Ebrahim, 2004), thus influencing the ability to infer the cause of disease. There was limited evidence of casual associations of Lp(a) with ischemic stroke and its subtypes from the UK Biobank and MEGASTROKE consortium databases. Casual associations of previous studies of Lipoprotein(a) with stroke are inconclusive (Chapman et al., 1998; Wang et al., 2022). Therefore, the causal connections between Lp(a) and stroke needs additional exploration.

Mendelian randomization (MR) follows the Mendelian genetic law of the “random distribution of parental alleles to offspring,” which is equivalent to random grouping in a randomized controlled trial (RCT) (Lawlor et al., 2008). Genetic variation that is strongly related to exposure can be used as a tool to evaluate the causal connections between exposure and outcomes (Lawlor et al., 2008). Since genetic variation is inherent, it meets the temporal requirement of causal inference and is not affected by environmental, social and other factors. Therefore, MR can effectively overcome the bias caused by confounding factors, and can provide reliable evidence for inferring causal relationships between exposure factors and outcomes.

Two sample Mendelian randomization is simple and easy to implement, providing a powerful statistical tool for causal inference in epidemiological observational study. The design strategy is to have two independent samples from the same population (such as GWAS and exposure, GWAS and outcome), requiring the two samples to have similar age, gender, and racial distribution characteristics. Due to the large sample size, this method can obtain greater confidence, with high confidence, economy, and efficiency, and can reduce the risk of bias caused by confounding factors and reverse causal relationships (Lawlor et al., 2008).

This study therefore used the two-sample MR method to evaluate the potential causal connection between Lp(a) and the risk of any stroke and specific stroke subtypes.

## 2. Materials and methods

### 2.1. Date sources

Summary statistical data of outcome, ischemic stroke and ischemic stroke subtypes were acquired from the opened genome-wide association study (GWAS), and the data of outcomes is conducted by MEGASTROKE consortium (Malik et al., 2018). The predominant outcomes for this MR study were stroke, ischemic stroke, and etiologic ischemic stroke subtypes (large-artery atherosclerotic stroke, small vessel stroke, and cardioembolic stroke), which are described by TOAST criteria (Wray et al., 2018). This study is based on the publicly available, large-scale GWAS summary datasets. All participants gave informed consent in all these corresponding original studies. The Ningbo First Hospital reviewed and approved this study (2021-R119).

The UK Biobank study is a community-based prospective cohort study carried out between 2006 and 2010 that contains in-depth genetic and health information from over 500,000 participants aged 40–69 years from different socioeconomic backgrounds at 22 health centers through the United Kingdom (Sudlow et al., 2015). Determination of stroke, ischemic stroke, and large-artery atherosclerotic stroke is based on the international Classification of Diseases ICD-10 codes I64, I63.9, and I63.2. The study passed the ethical review of the UK National Health Service National Research Ethics Service (Ref: 11/NW/0382 and 2018-1872). All participants in this study signed an informed consent form. Descriptive characteristics of the GWASs that were involved in the MR study are shown in Supplementary Table S1. After excluding those who had missing data on Lp(a) (*n* = 126,846), were non-European (*n* = 45,647), had prevalent stroke at baseline (*n* = 5,107), death at the time of the study (*n* = 23,759), and had missing data on covariates(*n* = 45,767)，a total of 255,286 individuals remained for the final analysis. The flow chart of study population was shown in Supplementary Figure S1.

### 2.2. Filtering of instrumental variables

We exploited data on genetic variants related with Lp(a) from the MEGASTROKE consortium and UK Biobank databases. In the first step, single-nucleotide polymorphisms (SNPs) achieved genome-wide significance (*p* < 5 × 10^−8^) to Lp(a) were pooled using genome-wide information from the European 1,000 Genomes Project as a criterion. The linkage disequilibrium (LD) parameter (*r*^2^) was set at a threshold of 0.001 and the genetic distance was set at 10 MB to select SNPs with the smallest *p* value, so as to ensure the independence between of the instrumental variables (IVs) and exclude the influence of LD on the results. In the second step, SNPs with genome-wide significance (*p* < 5 × 10^−8^) for stroke risk were excluded. The eight genetic variants used for selection of IVs for Lp(a) are shown in Supplementary Table S2.

### 2.3. Statistical analysis

We utilized the (inverse-variance weighted, IVW), weighted median method and MR-Egger method to analyze the main results of the MR (Nikolakopoulou et al., 2014; Bowden et al., 2016, 2017). The results of random-effect IVW method was regarded as the predominant analysis.

Cochran’s Q test was used to evaluate heterogeneity, and MR Egger’s intercept term was used to evaluate pleiotropy (Burgess and Thompson, 2017). The identification of heterogeneity in this study was done by two analytical methods, MR-IVW and MR-Egger analyses. The statistic for the MR-IVW analyses was *I*^2^ index and Cochran’s Q and the statistic for the MR-Egger analyses was Rucker’s Q (Hemani et al., 2018). This study utilized the MR-Egger method to evaluate the level of directional pleiotropy affected the hazard estimates from the intercept assessment (Verbanck et al., 2018). Additionally, we conducted a leave-one-out to evaluate the influence by every SNPs for sensitivity analysis. The MR analyses were calculated with the R software version 3.5.3 using the packages TwoSampleMR (Hemani et al., 2018) and MR-PRESSO (Verbanck et al., 2018).

Multivariate adjusted Cox proportional hazards models were also utilized to evaluate the connections between Lp(a) and stroke risk. These assessments were conducted using SAS software version 9.4 (SAS Institute Inc., Cary, NC, United States). A two-sided *p* value of less than 0.05 was deemed to denote statistical significance.

## 3. Results

The baseline clinical characteristics of all participants according LDL level were shown in Supplementary Table S3. We selected 8 independent SNPs as IVs for Lp(a). The essential information of these SNPs is described in Table 1. The distribution of F-statistics corresponding to a single SNP ranged from 186.82 to 1124.98, indicating that the causal correlations were less inclined to be affected by weak IV bias.

**Table 1:** The selected instrument variables of Lp(a).

SNP	EA	OA	*β*	SE	*p*-value	*F*
rs73596816	A	G	19.2	0.6	1.00E-200	1124.98
rs41266379	C	T	7.1	0.7	3.86E-24	317.92
rs143461353	T	C	13.1	1	4.09E-39	454.66
rs142126734	A	G	7.5	0.5	1.06E-50	454.66
rs41259144	T	C	−9.6	0.8	4.14E-33	385.56
rs41267809	G	A	−6.6	0.6	4.26E-28	317.92
rs34371670	T	C	−8.4	0.7	4.14E-33	385.56
rs139389770	G	T	−5.2	0.9	7.63E-09	186.82

SNPs, single nucleotide polymorphism; EA, effective allele; OA, non effective allele.

**Figure 1:**
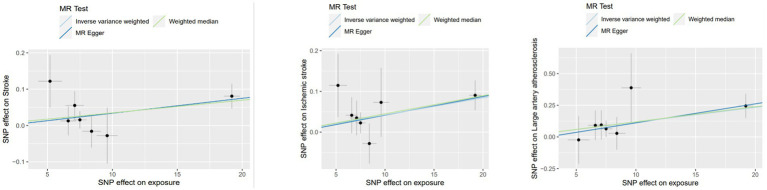
MR results for single instrument variable of Lp(a) on stroke and subtypes in MEGASTROKE.

The connections of the genetically determined hazard of Lp(a) with stroke and three ischemic stroke subtypes as derived using the two-sample MR method are shown in Table 2 and Figure 1. The causal estimates of the genetically determined risk of Lp(a) are displayed as ORs and 95% CI.

**Table 2:** Estimates the associations of Lp(a) and the Stroke and subtypes in MEGASTROKE from Mendelian randomization (MR) analysis.

Stroke	Method	OR	SE	P	95% Lower	95% Upper
Stroke	MR Egger	1.004	0.003	0.245	0.998	1.010
	Inverse variance weighted	1.003	0.001	0.010	1.001	1.006
	Weighted median	1.003	0.002	0.027	1.000	1.007
Ischemic stroke	MR Egger	1.005	0.003	0.230	0.998	1.011
	Inverse variance weighted	1.004	0.001	0.004	1.001	1.007
	Weighted median	1.004	0.002	0.007	1.001	1.008
Large artery atherosclerosis	MR Egger	1.015	0.009	0.145	0.998	1.033
	Inverse variance weighted	1.012	0.004	0.002	1.004	1.019
	Weighted median	1.012	0.004	0.005	1.004	1.020
Cardioembolic	MR Egger	1.009	0.007	0.239	0.996	1.022
	Inverse variance weighted	1.005	0.003	0.110	0.999	1.011
	Weighted median	1.006	0.003	0.056	1.000	1.013
Small-vessel	MR Egger	1.007	0.011	0.563	0.985	1.029
	Inverse variance weighted	1.003	0.005	0.479	0.994	1.012
	Weighted median	1.005	0.004	0.217	0.997	1.013

**Figure 2:**
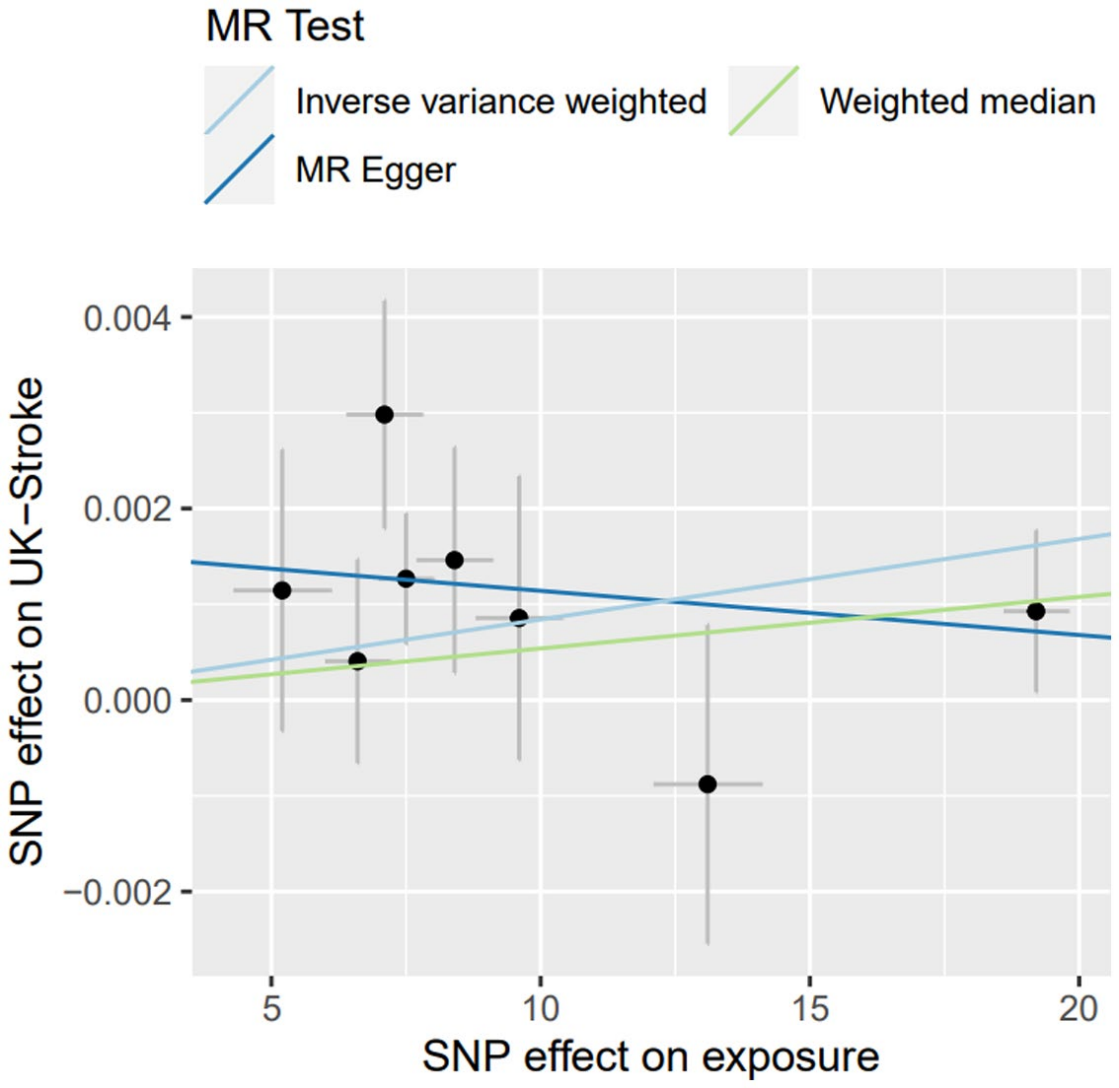
The sensitive analysis of Lp(a) on stroke in UK-biobank by MR.

Lp(a) was positively causally related to total stroke, ischemic stroke, and large-artery atherosclerotic stroke (Table 2). The ORs of total stroke, ischemic stroke, and large-artery atherosclerotic stroke of the genetically determined hazard of Lp(a) in MEGASTROKE in the predominant analysis were (1.003 [1.001–1.006], *p* = 0.010), (1.004 [1.001–1.007], *p* = 0.004), and (1.012 [1.004–1.019], *p* = 0.002), respectively. The weighted median in the sensitivity analysis had a consistent direction of estimation. In the primary analysis, the OR of total stroke in terms of the genetically determined risk of Lp(a) in the UK Biobank database was 1.001 [1.004–1.019], *p* = 0.017 (Table 3; Figure 2).

**Table 3:** Estimates the associations of Lp(a) and Stroke and subtypes in UK-biobank from Mendelian randomization (MR) analysis.

Method	OR	SE	*p*	95% Lower	95% Upper
MR Egger	1.000	7.80E-05	0.576	1.000	1.001
Inverse variance weighted	1.001	3.52E-05	0.017	1.001	1.002
Weighted median	1.000	4.01E-05	0.180	1.000	1.001

Compared with the total stroke hazard of the participants in the first quartile of Lp(a), a significantly higher hazard of total stroke was found among participants in the fourth quartile of Lp(a) in Model 2 (adjusted HR [95% CI], 0.93 [0.88–0.99], *p* = 0.006). A higher hazard of ischemic stroke was found for participants in the fourth quartile of Lp(a) in Model 4 (adjusted HR [95% CI], 0.93 [0.88–0.99], *p* < 0.001) (Table 4). The characteristics of the participants with different outcomes in the UK Biobank database are shown in Supplementary Table S4.

**Table 4:** Estimates the associations of Lp(a) and stroke and ischemic stroke in UK-biobank from observational analysis.

	Quartiles of Lp(a)				*p* for trend
	Q1	Q2	Q3	Q4	
Stroke					
Model 1	1	1.03 (0.95–1.11)	1.07 (0.991–1.156)	1.09 (1.03–1.18)*	0.013
Model 2	1	1.03 (0.96–1.12)	1.08 (1.00–1.17)*	1.11 (1.02–1.19)*	0.006
Ischemic stroke					
Model 3	1	1.05 (0.95–1.15)	1.09 (0.993–1.19)	1.16 (1.06–1.27)**	0.001
Model 4	1	1.06 (0.96–1.16)	1.11 (1.01–1.21)*	1.18 (1.08–1.29)**	<0.001
Hemorrhagic stroke					
Model 5	1	1.01 (0.86–1.17)	1.02 (0.87–1.18)	0.98 (0.84–1.14)	0.81
Model 6	1	1.02 (0.87–1.19)	1.04 (0.89–1.21)	1.00 (0.85–1.16)	0.95

Model 1 and Model 2: adjusted for gender, age, average total household income before tax, townsend deprivation index at recruitment, employment, SBP, DBP, smoke status, alcohol intake frequency, Waist/Hip circulation (all of these variables were statistically significant in the multivariable analysis). *p<0.005.

Model 3 and Model 4: additional adjustment for LDL, HDL, Triglycerides based on model 1 and model 2. **p≤0.001.

Model 5: adjusted for gender, age, average total household income before tax, employment, SBP, DBP, smoke status, alcohol intake frequency, Waist/Hip circulation (all of these variables were statistically significant in the multivariable analysis).

Model 6: additional adjustment for LDL based on model 5.

**Figure 3:**
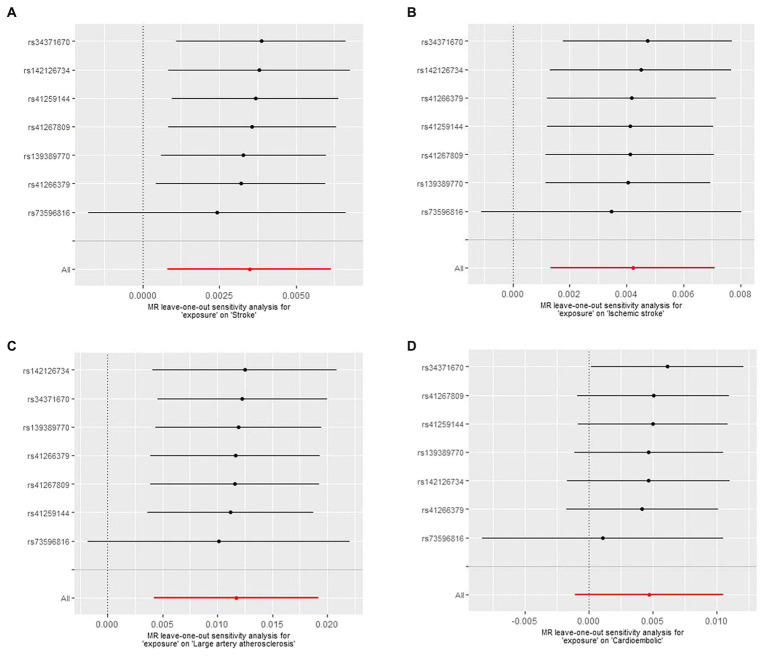
The Mendelian randomization (MR) leave-one-out sensitivity analysis for “exposure” on stroke and subtypes. **(A)** Stroke; **(B)** Ischemic stroke; **(C)** Large artery atherosclerosis; **(D)** Cardioembolic.

There was no manifestation of heterogeneity or pleiotropy of Lp(a) with outcomes in either the MEGASTROKE or UK Biobank databases (all *p* values >0.05) (Supplementary Tables S5, S6). MR results for single instrument variable of Lp(a) on Stroke and subtypes in MEGASTROKE and UK Biobank were shown in Supplementary Tables S7, S8, respectively. The results of the leave-one-out analysis denoted that no single variant impacted the connections between Lp(a) and stroke or its subtypes (Figure 3).

## 4. Discussion

The predominant MR analysis indicated that genetic proclivity to higher levels of Lp(a) was related with a higher hazard of total stroke, ischemic stroke, and large-artery atherosclerotic stroke. Higher levels of Lp(a) were also related with elevated stroke and ischemic stroke risk in the observational research data from the UK Biobank database.

Currently, the findings on the connection between Lp(a) and stroke are not entirely unity. Smolders et al. (2007) reviewed 31 observational studies between 1966 and 2006 and indicated that Lp(a) levels were higher among stroke patients in 23 case–control studies (OR [95% CI]: 2.39 [1.57–3.63]), and in 5 prospective cohort studies, people in the higher 1/3 of Lp(a) distribution were more likely to have a stroke than those in the lower 1/3 Lp(a) distribution (RR [95% CI], 1.22 [1.04–1.43]), but this research did not stratify transient ischemic attack, ischemic stroke, and hemorrhagic stroke. In 2015, Nave et al. (2015) pooled 9 prospective cohort studies and 11 case–control studies conducted between 2006 and 2014 and consummated that Lp(a) perhaps an independent hazard factor for ischemic stroke (RR [95% CI], 1.29 [1.06–1.58]; OR [95% CI], 1.41 [1.26–1.57]). However, a 2011 meta-analysis that included 16 prospective cohort studies did not find these risks (RR [95% CI], 1.12 [0.94–1.33], *p* = 0.137) (Genser et al., 2011). Lately, a meta-analysis from 2021 that included digest data from prospective and cross-sectional studies and reported standardized mean differences also indicated that an connection between high Lp(a) levels and elevated hazard of ischemic stroke, large-artery atherosclerotic stroke, and intracerebral hemorrhage (Kumar et al., 2021).

It is now believed that Lp(a) exerts its pathogenic effects mainly through the proatherogenic and fibrinolytic systems (Cho et al., 2013). Multiple mechanisms of action are involved in the proatherogenic process of Lp(a) molecules. Akin to LDL molecules, circulating Lp(a) molecules can be deposited directly in the arterial vessel wall, promoting foam cell production and exerting atherogenic effects. Lp(a) can also infiltrate into the subendothelial space and form oxidized phospholipids through the oxidation of polyunsaturated fatty acid residues. Oxidized Lp(a) molecules have been indicated to be related with the development of stroke and are thought to have a more potent atherogenic effect than Lp(a) (Ma et al., 2014). Lp(a) can also upregulate adhesion factor expression, promote inflammatory factor production, and increase vascular endothelial cell permeability and inflammatory cell accumulation, leading to abnormal endothelial cell function and proliferation, smooth muscle migration, and increased platelet adhesion and, ultimately, atherogenesis (Ferretti et al., 2018).

Recently, the American Heart Association released a scientific statement stating that upraised Lp(a) is an standalone and causal hazard factor for atherosclerotic cardiovascular disease (Reyes-Soffer et al., 2022). The statement notes that Lp(a) raise the risk of cardiovascular disease primarily through proatherogenic, prothrombotic, and proinflammatory processes, and that its levels are approximately 70% to ≥90% genetically determined, with factors such as diet and exercise having a minor impact. However, the definition of a high level of Lp(a) depends on the laboratory tests and units of measurement used, ethnicity, underlying disease in the population, and clinical characteristics, which makes it difficult to establish universal clinical thresholds.

The statement also noted that Lp(a) is a element of danger for aortic stenosis and a risk factor and latent therapeutic target for atherosclerotic cardiovascular disease (ASCVD) (Kronenberg et al., 2022). The statement concluded that Lp(a) levels are largely genetically determined (>90%). It recommended that adults should be tested for Lp(a) levels at a minimum once to distinguish those at high cardiovascular hazard. Lp(a) screening should be performed in the following scenarios: (i) incidence of early-onset ischemic stroke, (ii) family history of early-onset ASCVD, and (iii) high Lp(a) levels without other identifiable risk factors. Screening is also recommended for patients with familial hypercholesterolemia (FH), a family history of very high Lp(a), and a personal or family history of ASCVD.

According to current clinical guidelines, the core principle in the administration of patients with elevated Lp(a) is to reduce the overall ASCVD risk, including the control of concomitant clinically significant dyslipidemia of all kinds. For those at high hazard of cardiovascular disease, intensive low-density lipoprotein cholesterol (LDL-C)-lowering therapy is required in addition to lifestyle interventions (Kronenberg et al., 2022). Using the Third China National Stroke Registry (CNSR-III) cohort, one study aimed to investigate whether lower LDL-C and inflammation levels would reduce the hazard of stroke recurrence related with elevated Lp(a) in participants with ischemic stroke or transient ischemic attack. The findings showed that in patients with ischemic stroke or transient ischemic attack, elevated Lp(a) was significantly related with the hazard of stroke recurrence. In the context of secondary prevention, the risk of stroke recurrence due to elevated Lp(a) was significantly reduced when LDL-C or interleukin-6 levels were kept low (Xu et al., 2022). Novel Lp(a)-lowering therapies targeting the hepatic synthesis of apolipoprotein A [apo(a)] are in clinical trials, and the efficacy and safety of lipoprotein plasma exchange to wipe out Lp(a) and other apoB-containing lipoproteins is also under trial (Reyes-Soffer et al., 2022).

In this study, large-scale GWAS datasets and two-sample MR analyses were utilized to appraise the potential causal association between Lp(a) and stroke. The findings suggest a causal connection between Lp(a) and stroke from the perspective of genetics, providing a valuable reference for subsequent scientific research and new ideas for future clinical diagnosis, treatment, and prevention. Further, the GWAS datasets are of European origin, reducing the impact of population stratification on the potential associations and reducing the interference of confounding factors.

However, in practice, two sample MR analysis still faces many applications and methodological challenges. First, the two sample MRs (GWAS samples of exposure and outcome are required not to overlap each other), otherwise biased causal effect estimates will be generated. Second, GWAS populations of exposure and outcome should have similar ethnic characteristics, such as all from European or Asian populations. If two samples come from different populations, there may be bias in the estimation of causal effects (Lawlor et al., 2008).

Its contributions notwithstanding, this study also has some limitations. First, MR cannot fully explore the biological mechanism of genetic variation in Lp(a) (Burgess et al., 2014). Second, this study used two GWAS summary databases but no individual data, so it is impossible to conduct subgroup analyses by age or sex, for example, or to compare the differences in causal effects between subgroups.

In brief, our two-sample MR study suggests a probable causal impact of Lp(a) on the hazard of total stroke, ischemic stroke, and large-artery atherosclerotic stroke, highlighting the need for special attention to be paid to stroke patients with high Lp(a) levels.

## Data availability statement

Publicly available datasets were analyzed in this study. This data can be found here: Summary statistical data were acquired from the publicly genome-wide association study (GWAS), and MEGASTROKE consortium.

## Ethics statement

The studies involving human participants were reviewed and approved by The Ningbo First Hospital. The patients/participants provided their written informed consent to participate in this study.

## Author contributions

JS and XG: Conceptualization and Methodology. YH: Writing – Original Draft, Conceptualization, and Methodology. RZ: Formal analysis, Investigation, Data Curation, Writing – Original Draft, and Validation. LH: Conceptualization, Methodology, and Writing – Review & Editing. YiW: Validation, Formal analysis, Investigation, and Data Curation. XD, TX, and YuW: Validation and Investigation. All authors contributed to the article and approved the submitted version.

## Funding

This study was funded by the grants from the Zhejiang Provincial Natural Science Foundation of China (LY22H090001), Medicine and Health Science and Technology Projects of Zhejiang Province (2022KY305, 2022KY322), Ningbo Natural Science Foundation (2022J211, 2022J213), Ningbo Top Medical and Health Research Program (2022020304), Ningbo Medical and Health Brand Discipline (PPXK2018-04), and Key Laboratory of Precision Medicine for Atherosclerotic Diseases of Zhejiang Province (2022E10026).

## Conflict of interest

The authors declare that the research was conducted in the absence of any commercial or financial relationships that could be construed as a potential conflict of interest.

## Publisher’s note

All claims expressed in this article are solely those of the authors and do not necessarily represent those of their affiliated organizations, or those of the publisher, the editors and the reviewers. Any product that may be evaluated in this article, or claim that may be made by its manufacturer, is not guaranteed or endorsed by the publisher.
