# Supplementary material for: Lipoprotein(a) and stroke: a two-sample Mendelian randomization study
Source: Front Aging Neurosci. 2023 May 12;15:1178079. doi: 10.3389/fnagi.2023.1178079 (PMC10213338; doi:10.3389/fnagi.2023.1178079)
Supplement: Supplementary file 1 [file Table_5.docx]

Supplementary Material

Lipoprotein(a) and stroke: a two-sample Mendelian randomization study

Yi Huang^1,2,†^, Ruijie Zhang^3,4†^, Liyuan Han^3,4^, Yiwen Wu^1^, Xinpeng Deng^1^, Tianqi Xu^5^, Yuefei Wu^5^, Xiang Gao^1^, Chenhui Zhou^1,*^, Jie Sun^1,*^

*** Correspondence:**Chenhui Zhou and Jie Sun, Department of Neurosurgery, The First Affiliated Hospital of Ningbo University, Ningbo, Zhejiang 315010, China. Email: [fyyzhouchenhui@nbu.edu.cn](mailto:fyyzhouchenhui@nbu.edu.cn) (CZ), [fyysunjie@nbu.edu.cn](mailto:fyysunjie@nbu.edu.cn) (JS)

**^†^**These authors have contributed equally to this work and share first authorship

**Table S1.** Descriptive characteristics of the genome-wide association studies (GWAS) that were included in the Mendelian randomization study.

| **GWAS** | **Phenotype** | **Sample size** | **Ancestry** | **Adjustments** | **Use in this MR study** |
| --- | --- | --- | --- | --- | --- |
| MEGASTROKE | Any stroke, any ischemic stroke and subtypes (LAS, CES, SVS) | 67,162 cases/ 454,450 controls | Multi-ancestry | age, sex | Primary outcome in discovery analysis |
| UK Biobank | Any stroke | 4,985 cases/ 364,434 controls | European | age, sex, genotyping platform array | Primary outcome in validation analysis |

**Table S2.** The genetic variants used for selection of instrument variables for Lp(a)

| position | rsID | EA | OA | MAF | beta | se | t | p |
| --- | --- | --- | --- | --- | --- | --- | --- | --- |
| 6:160997118 | rs746173841 | T | A | 0.072 | 42.4 | 0.5 | 84.8 | 0.E+00 |
| 6:161013013 | rs1405708862 | C | T | 0.011 | 80.2 | 0.8 | 100.25 | 0.E+00 |
| 6:161017363 | rs73596816 | A | G | 0.034 | 19.2 | 0.6 | 32 | 2.E-221 |
| 6:160891897 | rs182443492 | A | C | 0.009 | 36.8 | 1 | 36.8 | 9.E-291 |
| 6:161032800 | rs369686024 | A | G | 0.014 | 19.2 | 0.8 | 24 | 3.E-126 |
| 6:161089307 | rs56393506 | T | C | 0.169 | 12.4 | 0.4 | 31 | 4.E-208 |
| 6:160831796 | rs151135411 | A | G | 0.001 | 69.5 | 2.7 | 25.74074 | 1.E-144 |
| 6:161292838 | rs145099029 | C | A | 0.003 | 17.8 | 1.8 | 9.888889 | 5.E-23 |
| 6:160998199 | rs41267813 | A | G | 0.001 | -58.8 | 2.9 | -20.2759 | 7.E-91 |
| 6:160890350 | rs6916433 | T | A | 0.14 | -4.7 | 0.3 | -15.6667 | 4.E-55 |
| 6:161137990 | rs783147 | A | G | 0.45 | -2.0 | 0.3 | -6.66667 | 3.E-11 |
| 6:160953137 | rs41266379 | C | T | 0.02 | 7.1 | 0.7 | 10.14286 | 4.E-24 |
| 6:160954800 | rs143461353 | T | C | 0.008 | 13.1 | 1 | 13.1 | 4.E-39 |
| 6:160942926 | rs142126734 | A | G | 0.049 | 7.5 | 0.5 | 15 | 1.E-50 |
| 6:160899049 | rs139609547 | ‐ | A | 0.054 | 4.4 | 0.4 | 11 | 4.E-28 |
| 6:161162290 | rs1835346 | G | A | 0.022 | 5.2 | 0.7 | 7.428571 | 1.E-13 |
| 6:161159366 | rs4252152 | G | T | 0.014 | 9.1 | 0.9 | 10.11111 | 5.E-24 |
| 6:161078894 | rs79246098 | C | T | 0.01 | 6.2 | 0.9 | 6.888889 | 6.E-12 |
| 6:160966559 | rs139145675 | A | G | 0.001 | -22.5 | 2.4 | -9.375 | 7.E-21 |
| 6:161022107 | rs41259144 | T | C | 0.011 | -9.6 | 0.8 | -12 | 4.E-33 |
| 6:161012805 | rs9456551 | C | T | 0.35 | 3.6 | 0.2 | 18 | 4.E-72 |
| 6:160953642 | rs41267809 | G | A | 0.022 | -6.6 | 0.6 | -11 | 4.E-28 |
| 6:161257953 | rs34371670 | T | C | 0.016 | -8.4 | 0.7 | -12 | 4.E-33 |
| 6:161070653 | rs41269876 | A | C | 0.028 | -8.2 | 0.6 | -13.6667 | 2.E-42 |
| 6:160909667 | rs141834709 | A | T | 0.009 | 8.7 | 1 | 8.7 | 3.E-18 |
| 6:161162406 | rs4252170 | C | T | 0.082 | 3.2 | 0.4 | 8 | 1.E-15 |
| 6:161251940 | rs138491411 | G | A | 0.012 | 5.0 | 0.8 | 6.25 | 4.E-10 |
| 6:160720804 | rs183815886 | C | G | 0.003 | 14.8 | 1.8 | 8.222222 | 2.E-16 |
| 6:160847571 | rs117446263 | A | G | 0.022 | -5.2 | 0.6 | -8.66667 | 5.E-18 |
| 6:160543317 | rs200684404 | T | C | 0 | 67.7 | 9.2 | 7.358696 | 2.E-13 |
| 6:160493099 | rs200144324 | T | C | 0 | 81.5 | 11.3 | 7.212389 | 6.E-13 |
| 6:161087652 | rs77337569 | G | T | 0.013 | 5.2 | 0.8 | 6.5 | 8.E-11 |
| 6:161214526 | rs186418835 | A | G | 0.004 | -9.7 | 1.5 | -6.46667 | 1.E-10 |
| 6:161177443 | rs117534432 | T | C | 0.036 | 3.3 | 0.5 | 6.6 | 4.E-11 |
| 6:161011999 | rs200376184 | C | G | 0.001 | 17.5 | 2.7 | 6.481481 | 9.E-11 |
| 6:161189071 | rs11753588 | A | G | 0.109 | -2.4 | 0.3 | -8 | 1.E-15 |
| 6:161285760 | rs4709474 | G | A | 0.49 | 1.7 | 0.2 | 8.5 | 2.E-17 |
| 6:161031132 | rs191690882 | A | G | 0.002 | -13.2 | 1.9 | -6.94737 | 4.E-12 |
| 6:161255668 | rs182349273 | G | A | 0 | 34.4 | 5.7 | 6.035088 | 2.E-09 |
| 6:161088956 | rs75274517 | A | G | 0.01 | -6.5 | 1 | -6.5 | 8.E-11 |
| 6:160825930 | rs143365644 | T | A | 0.035 | 3.7 | 0.5 | 7.4 | 1.E-13 |
| 6:161135746 | rs139389770 | G | T | 0.011 | -5.2 | 0.9 | -5.77778 | 8.E-09 |
| 6:161250301 | rs140606700 | G | A | 0.007 | 6.4 | 1.2 | 5.333333 | 1.E-07 |

**Table S3**. Characteristics of the study population according to the Lp(a) quartiles

| Variables |  | Quatile 1  (<4.88 nmol/L) | Quatile 1  (4.88-6.15 nmol/L) | Quatile 1  (6.15-7.66 nmol/L) | Quatile 1  (>7.66 nmol/L) | Statistic test | P-value |
| --- | --- | --- | --- | --- | --- | --- | --- |
| Gender,n(%) | Female | 31236(23.54%) | 33141(24.97%) | 35094(26.44%) | 33240(25.05%) | 444.332 | <0.001 |
|  | Male | 32219(26.29%) | 31203(25.46%) | 28622(23.35%) | 30531(24.91%) |  |  |
| Income,n(%) | <18000£ | 13519(24.24%) | 13743(24.64%) | 14580(26.14%) | 13938(24.99%) | 145.251 | <0.001 |
|  | 18000-30999£ | 15933(24.39%) | 16296(24.94%) | 16751(25.64%) | 16352(25.03%) |  |  |
|  | 31000-51999£ | 16948(25.00%) | 17295(25.51%) | 16681(24.60%) | 16879(24.89%) |  |  |
|  | 52000-100000£ | 13524(25.66%) | 13459(25.54%) | 12449(23.62%) | 13265(25.17%) |  |  |
|  | >100000£ | 3531(25.82%) | 3551(25.97%) | 3255(23.80%) | 3337(24.40%) |  |  |
| Current employment status,n(%) | In paid employment or self-employed | 39075(25.37%) | 39110(25.40%) | 37050(24.06%) | 38761(25.17%) | 255.372 | <0.001 |
|  | Retired | 19732(23.63%) | 20792(24.90%) | 22293(26.70%) | 20685(24.77%) |  |  |
|  | Looking after home and/or family | 1561(26.03%) | 1496(24.95%) | 1460(24.35%) | 1480(24.68%) |  |  |
|  | Unable to work because of sickness or disability | 1828(26.32%) | 1710(24.62%) | 1757(25.30%) | 1651(23.77%) |  |  |
|  | Unemployed | 899(26.71%) | 861(25.58%) | 800(23.77%) | 806(23.95%) |  |  |
|  | Doing unpaid or voluntary work | 244(24.80%) | 251(25.51%) | 243(24.70%) | 246(25.00%) |  |  |
|  | Others | 116(23.43%) | 124(25.05%) | 113(22.83%) | 142(28.69%) |  |  |
| Smoke,n(%) | Never | 24830(24.70%) | 25479(25.34%) | 25146(25.01%) | 25081(24.95%) | 3.205 | <0.001 |
|  | Ever/Current | 38625(24.96%) | 38865(25.11%) | 38570(24.92%) | 38690(25.00%) |  |  |
| Alcohol intake frequency,n(%). | Daily or almost daily | 14434(26.07%) | 13835(24.99%) | 13341(24.10%) | 13756(24.85%) | 83.256 | <0.001 |
|  | Three or four times a week | 15561(24.71%) | 15938(25.31%) | 15578(24.74%) | 15893(25.24%) |  |  |
|  | Once or twice a week | 16501(24.54%) | 17115(25.46%) | 16948(25.21%) | 16668(24.79%) |  |  |
|  | One to three times a month | 6919(24.42%) | 7121(25.13%) | 7246(25.57%) | 7051(24.88%) |  |  |
|  | Special occasions only | 6246(24.19%) | 6460(25.02%) | 6644(25.73%) | 6470(25.06%) |  |  |
|  | Never | 3794(24.38%) | 3875(24.90%) | 3959(25.44%) | 3933(25.27%) |  |  |
| Waist/Hip,median (Q25,Q75) | | 0.880(0.806,0.942) | 0.875(0.804,0.937) | 0.870(0.802,0.932) | 0.874(0.802,0.938) | 236.714 | <0.001 |
| Townsend deprivation index,median (Q25,Q75) | | -2.313(-3.719,0.123) | -2.354(-3.752,0.021) | -2.350(-3.734,0.051) | -2.337(-3.742,0.046) | 10.549 | 0.014 |
| LDL cholesterol, median (Q25,Q75) | | 3.407(2.859,4.002) | 3.507(2.947,4.083) | 3.614(3.050,4.200) | 3.592(3.024,4.183) | 2039.544 | <0.001 |
| HDL cholesterol, median (Q25,Q75) | | 1.383(1.154,1.665) | 1.394(1.170,1.666) | 1.409(1.187,1.676) | 1.401(1.175,1.675) | 170.37 | <0.001 |
| Triglycerides, cholesterol | | 1.534(1.067,2.253) | 1.494(1.053,2.169) | 1.480(1.056,2.103) | 1.443(1.021,2.093) | 376.354 | <0.001 |
| DBP, median (Q25,Q75) | | 82.50(76.00,90.50) | 82.50(75.50,90.50) | 82.50(75.50,90.50) | 82.50(75.50,90.50) | 2.789 | 0.425 |
| SBP, median (Q25,Q75) | | 135.00(122.50,148.50) | 134.50(122.00,148.00) | 135.00(122.50,148.50) | 134.50(122.00,148.00) | 17.811 | <0.001 |
| Age, median (Q25,Q75) | | 57(49,63) | 57(50,63) | 58(51,63) | 58(50,63) | 439.564 | <0.001 |

**Table S4.** The characteristics of participants for different outcomes in the UK Biobank

| Variables |  | Stroke | | | Ischemic Stroke | | | Hemorrhagic stroke | | |
| --- | --- | --- | --- | --- | --- | --- | --- | --- | --- | --- |
|  |  | No(n=249831) | Yes(n=5455) | P-value | No(n=251339) | Yes(n=3947) | P-value | No(n=253944) | Yes(n=1342) | P-value |
| Gender,n(%) | Female | 130569( 52.26% ) | 2142( 39.27% ) | <0.001 | 131282( 52.23% ) | 1429( 32.42% ) | <0.001 | 132056( 52.00% ) | 655( 48.81% ) | 0.019 |
|  | Male | 119262( 47.74% ) | 3313( 60.73% ) |  | 120057( 47.77% ) | 2518( 57.12% ) |  | 121888( 48.00% ) | 687( 51.19% ) |  |
| Income,n(%) | <18000£ | 53951( 21.59% ) | 1829( 33.53% ) |  | 54381( 21.64% ) | 1399( 31.74% ) |  | 55365( 21.80% ) | 415( 30.92% ) |  |
|  | 18000-30999£ | 63728( 25.51% ) | 1604( 29.40% ) |  | 64139( 25.52% ) | 1193( 27.06% ) |  | 64951( 25.58% ) | 381( 28.39% ) |  |
|  | 31000-51999£ | 66651( 26.68% ) | 1152( 21.12% ) |  | 67003( 26.66% ) | 800( 18.15% ) |  | 67503( 26.58% ) | 300( 22.35% ) |  |
|  | 52000-100000£ | 51990( 20.81% ) | 707( 12.96% ) |  | 52251( 20.79% ) | 446( 10.12% ) |  | 52492( 20.67% ) | 205( 15.28% ) |  |
|  | >100000£ | 13511( 5.41% ) | 163( 2.99% ) |  | 13565( 5.40% ) | 109( 2.47% ) |  | 13633( 5.37% ) | 41( 3.06% ) |  |
| Current employment status,n(%) | In paid employment or self-employed | 151775( 60.75% ) | 2221( 40.71% ) | <0.001 | 152467( 60.66% ) | 1529( 34.69% ) | <0.001 | 153422( 60.42% ) | 574( 42.77% ) | <0.001 |
|  | Retired | 80651( 32.28% ) | 2851( 52.26% ) |  | 81360( 32.37% ) | 2142( 48.59% ) |  | 82829( 32.62% ) | 673( 50.15% ) |  |
|  | Looking after home and/or family | 5941( 2.38% ) | 56( 1.03% ) |  | 5966( 2.37% ) | 31( 0.70% ) |  | 5975( 2.35% ) | 22( 1.64% ) |  |
|  | Unable to work because of sickness or disability | 6708( 2.69% ) | 238( 4.36% ) |  | 6772( 2.69% ) | 174( 3.95% ) |  | 6891( 2.71% ) | 55( 4.10% ) |  |
|  | Unemployed | 3299( 1.32% ) | 67( 1.23% ) |  | 3311( 1.32% ) | 55( 1.25% ) |  | 3351( 1.32% ) | 15( 1.12% ) |  |
|  | Doing unpaid or voluntary work | 964( 0.39% ) | 20( 0.37% ) |  | 969( 0.39% ) | 15( 0.34% ) |  | 982( 0.39% ) | 2( 0.15% ) |  |
|  | Others | 493( 0.20% ) | 2( 0.04% ) |  | 494( 0.20% ) | 1( 0.02% ) |  | 494( 0.19% ) | 1( 0.07% ) |  |
| Smoke,n(%) | Never | 98704( 39.51% ) | 1832( 33.58% ) | <0.001 | 99254( 39.49% ) | 1282( 29.08% ) | <0.001 | 100081( 39.41% ) | 455( 33.90% ) | <0.001 |
|  | Ever/Current | 151127( 60.49% ) | 3623( 66.42% ) |  | 152085( 60.51% ) | 2665( 60.46% ) |  | 153863( 60.59% ) | 887( 66.10% ) |  |
| Alcohol intake frequency,n(%). | Daily or almost daily | 54033( 21.63% ) | 1333( 24.44% ) | <0.001 | 54415( 21.65% ) | 951( 21.57% ) | <0.001 | 55031( 21.67% ) | 335( 24.96% ) | <0.001 |
|  | Three or four times a week | 61808( 24.74% ) | 1162( 21.30% ) |  | 62126( 24.72% ) | 844( 19.15% ) |  | 62696( 24.69% ) | 274( 20.42% ) |  |
|  | Once or twice a week | 65903( 26.38% ) | 1329( 24.36% ) |  | 66281( 26.37% ) | 951( 21.57% ) |  | 66897( 26.34% ) | 335( 24.96% ) |  |
|  | One to three times a month | 27821( 11.14% ) | 516( 9.46% ) |  | 27931( 11.11% ) | 406( 9.21% ) |  | 28224( 11.11% ) | 113( 8.42% ) |  |
|  | Special occasions only | 25194( 10.08% ) | 626( 11.48% ) |  | 25371( 10.09% ) | 449( 10.19% ) |  | 25657( 10.10% ) | 163( 12.15% ) |  |
|  | Never | 15072( 6.03% ) | 489( 8.96% ) |  | 15215( 6.05% ) | 346( 7.85% ) |  | 15439( 6.08% ) | 122( 9.09% ) |  |
| Waist/Hip,median (Q_25_,Q_75_) | | 0.88(0.81,0.94) | 0.91(0.84,0.97) | <0.001 | 0.88(0.81,0.94) | 0.92(0.85,0.98) | <0.001 | 0.88(0.81,0.94) | 0.89(0.82,0.95) | <0.001 |
| Townsend deprivation index,median (Q_25_,Q_75_) | | -2.25(-3.66,0.24) | -2.00(-3.48,0.73) | <0.001 | -2.25(-3.66,0.24) | -1.92(-3.43,0.9) | <0.001 | -2.25(-3.66,0.26) | -2.17(-3.53,-0.02) | 0.232 |
| LDL cholesterol, median (Q_25_,Q_75_) | | 3.54(2.96,4.13) | 3.43(2.81,4.07) | <0.001 | 3.54(2.96,4.13) | 3.4(2.76,4.04) | <0.001 | 3.54(2.96,4.13) | 3.42(2.83,4.08) | <0.001 |
| HDL cholesterol, median (Q_25_,Q_75_) | | 1.38(1.16,1.65) | 1.3(1.09,1.55) | <0.001 | 1.38(1.16,1.65) | 1.27(1.07,1.52) | <0.001 | 1.38(1.16,1.65) | 1.38(1.15,1.65) | 0.116 |
| Lipoprotein A, median (Q_25_,Q_75_) | | 20.36(9.38,60.2) | 21.25(9.51,62.82) | 0.027 | 20.36(9.38,60.2) | 21.63(9.5,64.12) | 0.006 | 20.4(9.39,60.24) | 20.05(9.5,55.51) | 0.853 |
| Triglycerides, cholesterol | | 1.53(1.08,2.21) | 1.64(1.18,2.33) | <0.001 | 1.53(1.08,2.21) | 1.69(1.2,2.39) | <0.001 | 1.54(1.08,2.21) | 1.51(1.05,2.2) | 0.433 |
| DBP, median (Q_25_,Q_75_) | | 83(76,90.5) | 85(77.5,92.5) | <0.001 | 83(76,90.5) | 84.75(77.5,92.5) | <0.001 | 83(76,90.5) | 83.75(77,92) | <0.001 |
| SBP, median (Q_25_,Q_75_) | | 136(123,149.5) | 142.5(128.5,156.5) | <0.001 | 136(123,149.5) | 143(129.5,157) | <0.001 | 136(123.5,149.5) | 141(127.5,154.5) | <0.001 |
| Age, median (Q_25_,Q_75_) | | 59(51,64) | 64(59,67) | <0.001 | 59(51,64) | 64(59,67) | <0.001 | 59(51,64) | 63(58,67) | <0.001 |

**Table S5.** The heterogeneity tests for instrument variable of Lp(a)

|  | Method | Q | Q_df | Q_pval |
| --- | --- | --- | --- | --- |
| Stroke | IVW | 4.798 | 6 | 0.570 |
| Ischemic stroke | IVW | 3.568 | 6 | 0.735 |
| Large artery atherosclerosis | IVW | 1.737 | 6 | 0.942 |
| Cardioembolic | IVW | 4.618 | 6 | 0.594 |
| Small-vessel | IVW | 10.538 | 6 | 0.104 |
| Stroke(UK) | IVW | 4.245 | 7 | 0.751 |

**Table S6.** Pleiotropy tests for MR analysis

|  | Egger_intercept | SE | P value |
| --- | --- | --- | --- |
| Stroke | -0.007 | 0.033 | 0.831 |
| Ischemic stroke | -0.005 | 0.036 | 0.904 |
| Large artery atherosclerosis | -0.039 | 0.092 | 0.690 |
| Cardioembolic | -0.0509 | 0.072 | 0.513 |
| Small-vessel | -0.044 | 0.117 | 0.726 |
| Stroke(UK) | 0.001 | 0.001 | 0.212 |

**Table S7.** MR results for single instrument variable of Lp(a) on Stroke and subtypes in MEGASTROKE.

| SNP | Stroke | | Ischemic stroke | | Large artery atherosclerosis | | Cardioembolic | | Small-vessel | |
| --- | --- | --- | --- | --- | --- | --- | --- | --- | --- | --- |
|  | OR | 95%CI | OR | 95%CI | OR | 95%CI | OR | 95%CI | OR | 95C%CI |
| rs139389770 | 1.024 | 0.996~1.052 | 1.022 | 0.992~1.052 | 0.996 | 0.927~1.068 | 1.007 | 0.951~1.065 | 0.981 | 0.923~1.042 |
| rs142126734 | 1.002 | 0.996~1.008 | 1.003 | 0.996~1.009 | 1.009 | 0.991~1.025 | 1.005 | 0.990~1.019 | 1.012 | 0.997~1.027 |
| rs34371670 | 0.998 | 0.988~1.009 | 0.997 | 0.985~1.008 | 1.003 | 0.973~1.033 | 0.984 | 0.962~1.006 | 0.969* | 0.944~0.994 |
| rs41259144 | 0.997 | 0.982~1.013 | 1.008 | 0.990~1.025 | 1.041 | 0.985~1.100 | 0.995 | 0.960~1.029 | 1.022 | 0.985~1.060 |
| rs41266379 | 1.008 | 0.997~1.019 | 1.005 | 0.993~1.016 | 1.013 | 0.981~1.046 | 1.015 | 0.989~1.041 | 1.002 | 0.973~1.031 |
| rs41267809 | 1.002 | 0.990~1.014 | 1.006 | 0.993~1.019 | 1.014 | 0.979~1.049 | 0.999 | 0.973~1.024 | 0.993 | 0.964~1.022 |
| rs73596816 | 1.004^*^ | 1.001~1.008 | 1.005^*^ | 1.000~1.008 | 1.013^***^ | 1.003~1.022 | 1.007 | 0.999~1.014 | 1.005 | 0.995~1.013 |
| All - Inverse variance weighted | 1.003^*^ | 1.001~1.006 | 1.004^**^ | 1.001~1.007 | 1.012^***^ | 1.004~1.019 | 1.005 | 0.998~1.010 | 1.003 | 0.994~1.012 |
| All-MR Egger | 1.004 | 0.998~1.010 | 1.005 | 0.998~1.011 | 1.015 | 0.997~1.032 | 1.009 | 0.995~1.022 | 1.007 | 0.985~1.029 |

Note: *,**,***present p<0.05,0.01,0.001

**Table S8.** Estimates the associations of Lp(a) and Stroke and subtypes in UK-biobank from Mendelian randomization (MR) analysis.

| SNP | OR | SE | P | 95%CI |
| --- | --- | --- | --- | --- |
| rs139389770 | 1.000 | 2.82E-04 | 0.436 | 0.999-1.007 |
| rs142126734 | 1.000 | 9.10E-05 | 0.063 | 0.999-1.003 |
| rs143461353 | 1.000 | 1.27E-04 | 0.597 | 0.999-1.001 |
| rs34371670 | 1.002 | 1.40E-04 | 0.214 | 0.999-1.004 |
| rs41259144 | 1.001 | 1.54E-04 | 0.563 | 0.999-1.003 |
| rs41266379 | 1.004 | 1.67E-04 | 0.012 | 1.000-1.007 |
| rs41267809 | 1.001 | 1.61E-04 | 0.703 | 0.999-1.003 |
| rs73596816 | 1.000 | 4.40E-05 | 0.272 | 0.999-1.001 |
| All - Inverse variance weighted | 1.001 | 2.82E-04 | 0.017 | 1.000-1.001 |
| All - MR Egger | 1.000 | 9.10E-05 | 0.576 | 0.999-1.001 |


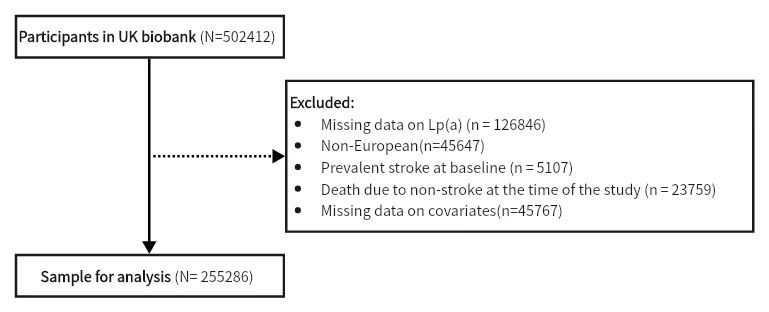


Fig.S1 The flow chart of study population
